# Decoloniality impact assessment for AI

**DOI:** 10.1007/s00146-025-02649-4

**Published:** 2025-09-28

**Authors:** Damian Eke, Ricardo Chavarriaga, Bernd Stahl

**Affiliations:** 1 School of Computer Science, University of Nottingham, Nottingham, UK; 2 Centre for Artificial Intelligence, Zurich University of Applied Sciences, Winterthur, Switzerland

**Keywords:** AI, Impact assessment, Decoloniality, Coloniality, Global South, Ethics, Responsibility

## Abstract

In the last decade, several organisations, and national and international agencies have developed impact assessments (IAs) to mitigate the risks and impact of AI systems as well as to promote responsible, just and trustworthy design, development and deployment. However, through a critical review of current AI IAs, we identify the failure of these IAs to address fundamental questions regarding who defines problems, whose knowledge is valued, and who truly benefits from AI innovation or generally what we term the ‘coloniality problem’. Developed primarily within Global North normative frameworks, these IAs risk perpetuating the very inequalities they aim to address by neglecting Global South perspectives and the extractive logic underpinning data practices. Thus, we propose a novel approach: Decoloniality Impact Assessment (DIA) as a critical, context-sensitive evaluative approach that assesses AI systems in relation to their inherent colonial legacies, global power asymmetries, and epistemic injustices. It moves beyond traditional ethical frameworks by interrogating how the AI innovation lifecycle and practices reinforce structural inequalities, marginalise local knowledge systems, and perpetuate exploitative systems. The paper advocates for an AI innovation lifecycle approach to DIA, recognising that coloniality manifests at every stage of AI development, from ideation to deployment. DIA is not a new impact assessment framework but an approach that can be integrated into already existing frameworks such as the Council of Europe’s HUDERIA framework. It is a call to reframe AI innovation in a way that technological futures are rooted in justice, pluriversality, and sovereignty.

## Introduction

In literature and in practice, Artificial Intelligence (AI) has been shown to revolutionise many aspects of daily life; from healthcare and education to agriculture, transportation, finance, and public services (Jiang et al. 2017; Bannerjee et al. 2018; Abduljabbar et al. 2019; Shaheen 2021). However, alongside these transformative potentials, AI raises numerous ethical, legal, socio-cultural, economic, and environmental concerns (Jobin et al. 2019; Crawford 2021). These include issues, such as discrimination (Buolamwini and Gebru 2018), privacy violations (Elliott and Soifer 2022), labour displacement (Yeh et al. 2020), the opaque nature of its decision-making (Smith 2021), and issues related to coloniality (Eke and Ogoh 2022; Wakunuma et al. 2025). In response, there has been a proliferation of AI impact assessment (IA) tools, designed to identify, anticipate, and mitigate risks. These tools aim at enhancing accountability, guide more responsible development and deployment of AI technologies, and ensure trustworthiness (Stahl et al. 2023).

Yet, despite their growing adoption, most existing AI impact assessments do not address the enduring issue of coloniality; that is, the continued colonial power relations and tendencies, epistemic hierarchies and injustices, and structural inequalities embedded in global AI knowledge production and technological design (Ruttkamp-Bloem 2023; Wakunuma et al. 2025). Not surprisingly, echoing the reality of technological development, many of these IA tools are developed within the Global North legal and ethical frameworks and are often exported to other contexts with little attention to the historical, political, and socio-cultural dynamics of the Global South. As a result, these assessments risk reproducing the very inequalities they seek to mitigate; by overlooking local perspectives, prioritising Western and or Chinese values, and failing to interrogate the extractive logic underlying data practices and AI development (Wakunuma et al. 2025; Eke et al. 2025). In this paper, we therefore explore the research question: How do existing AI impact assessment tools address social, ethical, and political concerns, and to what extent do they engage with structural and decolonial critiques?

This paper is the first to address this decoloniality lacuna in existing IAs and provide a methodologically robust account of what a Decoloniality Impact Assessment (DIA) can provide and a practical set of suggestions of how it can be implemented or integrated into existing IAs. Whereas there is an emerging literature on coloniality in the AI innovation lifecycle, this is the first attempt at addressing it via impact assessment frameworks. This work is of high intellectual relevance to the communities of scholars and practitioners who are interested in ethical, legal, and related aspects of AI and aim to understand current and future governance structures. The article is furthermore of high practical relevance, because it points to ways of improving current and emerging IAs at the exact time when these are starting to be rolled out and integrated into AI oversight and governance, for example through the EU’s AI Act, the fundamental rights Impact Assessment (FRIA), the UN’s human rights due diligence, or the Council of Europe’s adoption of the HUDERIA methodology.

This neglect of historical and normative frameworks from the Global South means that AI systems can be and are being developed and deployed in ways that reinforce colonial tendencies, and technological dependency, especially in Africa and other marginalised contexts. Through a critical analysis of existing AI IAs, this paper finds that although impact assessments regularly refer to stakeholder identification and engagement, they rarely ask who defines the problem, whose knowledge counts, or who benefits from the innovation. It then expands the scope of IAs (especially ones conducted in the Global South) to include decoloniality; recognising the ways AI technologies can replicate or exacerbate colonial legacies of socio-technical control, marginalisation, and power imbalances. This paper progresses with an overview of the ‘coloniality problem’ in AI; then provides details of the methodological choices, findings, and discussions; and concludes with a crucial discussion on a new impact assessment approach for AI based on decoloniality.

## AI, the coloniality problem and decoloniality

The concept of *coloniality* refers to the continuation of colonial tendencies, legacies, and structures of power, knowledge, and being after the formal end of colonisation (Quijano 2000; Maldonado-Torres 2007; Ndlovu‐Gatsheni 2015). According to Mohamed et al. (2020), coloniality refers to ‘‘the continuity of established patterns of power between coloniser and colonised—and the contemporary remnants of these relationships—and how that power shapes our understanding of culture, labour, intersubjectivity and knowledge production’’. This is an idea that has been highlighted in many shades by African scholars, such as Kwame Nkrumah (1965), Ngugi wa Thiong’o (1986), Chinweizu, (1975), Chinweizu and Madubuike (1983), as well as scholars from other parts of the Global South (Tlostanova and Mignolo 2009; Dube 2016; Subramaniam 2017; Bhatia 2021; Serrano-Muñoz 2021). Nkrumah conceptualised it as neo-colonialism which manifests in different ways and in different domains. In the context of AI, coloniality manifests in multiple interlinked areas (Muldoon and Wu 2023; Hao et al. 2024; Wakunuma et al. 2025), raising important questions about whose knowledge shapes AI, who benefits from it, and who is neglected, made invisible or vulnerable by it? This is what is referred to as the ‘coloniality problem’ in this paper.

One major area of AI’s coloniality lies in technological development and global power asymmetries. The design, governance, and regulation of AI are overwhelmingly dominated by a few actors in the Global North, particularly the US, China, and a few European countries (Eke et al. 2023a; b). The interesting thing here is that whilst China was historically colonised, it has been claimed that she has become one of the symbols of continued colonial tendencies in the Global south (Gravett 2023; Masigan 2024). Big tech companies, standard-setting bodies, and key research institutions that shape AI futures are in the Global North and China. This centralisation of influence results in epistemic monopolies (Ruttkamp-Bloem 2023). As a result, global frameworks for “ethical AI” or “responsible innovation” are developed within the Global North normative paradigms, with limited or no perspectives from African, indigenous, and other marginalised epistemic communities of the Global South such as countries in Latin America. These countries are often positioned as passive tech receivers or testing grounds for AI technologies, rather than active co-creators of AI.

Coloniality is also deeply embedded in AI’s dependence on large-scale data extraction, often from people, communities, and regions with little or no say in how their data are used (Couldry and Mejias 2019). Fundamentally, this is often supported by the uncritical framing of data as a neutral resource which then masks the exploitative dynamics at play in many data collection practices. Personal, biometric, health, linguistic, and environmental data are routinely harvested from African populations through health apps, social media, surveillance technologies, and development programmes without adequate informed consent, transparency, or equitable benefit-sharing (Mann 2018; Calzati 2022; Mano and Mukhongo 2025; Sarku and Ayamga 2025). This phenomenon has been described as *data colonialism* (Couldry & Mejias 2019), whereby data from the Global South are mined for digital value under the guise of innovation or development.

In terms of infrastructure, the global AI ecosystem is sustained by extractive practices that disproportionately affect resource-rich but economically disadvantaged regions, particularly in Africa (Okoi 2025). The demand for computational power and storage required by AI systems relies on minerals such as cobalt, lithium, and rare-earth elements (Goodenough et al. 2018), many of which are sourced from developing or least developed countries under exploitative labour and environmental conditions. The extractivism that underpinned the colonial economy thus finds new expression here, reinforcing patterns of environmental degradation and economic dependency.

A further manifestation of coloniality related to the above is in the labour dynamics underpinning data annotation and content moderation, where workers across several countries are employed as low-cost labour for the global AI supply chain (Regilme 2024; Adams 2024). Companies outsourcing data labelling tasks, such as image tagging, text annotation, or audio transcription, often recruit workers in the Global South through intermediary platforms or outsourcing firms (Okolo and Tano 2024). These workers are essential to the functionality and accuracy of AI systems, yet their labour is largely invisible and grossly undercompensated, often paid below living wages with limited labour protections, job security, or career advancement pathways. Investigations have revealed that workers are sometimes subjected to psychologically distressing tasks, such as moderating violent or explicit content, without access to mental health support (Steiger et al. 2021; Rowe 2023; Gupta 2024). This reproduces extractive colonial logics by commodifying African labour for the benefit of powerful AI firms based in the Global North, whilst maintaining a global division of cognitive and economic value creation.

The absence of African voices and expertise in global AI development teams exacerbates the coloniality problem (Ndaka et al. 2024). Despite the wealth of intellectual talent across the continent, African researchers are vastly underrepresented in key spaces of AI innovation, from academic publishing and standard-setting bodies to leadership in global tech firms. This exclusion is not only a matter of fairness but also leads to harmful consequences, as AI systems built without cultural or contextual understanding may reinforce stereotypes, fail to address local needs, or produce discriminatory outcomes.

There is also a growing body of literature focussing on “algorithmic colonialism” (Birhane 2020; Mohamed et al. 2020), which captures how AI systems are deployed in ways that replicate coercive or paternalistic relationships reminiscent of colonial governance. For example, AI tools used for content moderation or humanitarian interventions in African contexts can be embedded with foreign assumptions about governance, security, and morality, whilst displacing or overriding indigenous approaches to justice, health, or social cohesion.

In sum, the coloniality problem in AI is not merely a symbolic concern but a structural condition, one that shapes who builds AI, who it serves, and whose ways of knowing are legitimised or erased in the process. Addressing this issue demands a decolonial turn in AI governance: one that centres marginalised agency, supports local innovation ecosystems, reconfigures power in data governance, and recognises colonial histories as active forces shaping present AI imaginaries and infrastructures. This decolonial approach is best referred to as decoloniality (Wakunuma et al. 2025). This is different from anti-colonialism or decolonisation. Decoloniality is ‘‘an active, transformative, and interventional process…that involves identifying and addressing current colonial tendencies’’ (Wakunuma et al. 2025). Wakunuma et al. (2025) use the analogy of a stranger in the house to describe decolonisation and decoloniality where decolonisation is making sure that the stranger leaves, whilst decoloniality refers to the process of cleaning your house in the aftermath of a stranger leaving the house in ruin; including repairing the damages, renovating the house to your needs, taste, interests. They also framed decoloniality as an important requirement for trustworthy AI in the Global South. To this effect, it is imperative to develop practical pathways of achieving this requirement.

### Emerging decolonial practices as counters to coloniality

In the last few years, there are emerging practices aimed at reclaiming agency, sovereignty, and contextual relevance which are counters to coloniality in technology, data, and knowledge systems. Initiatives such as Masakhane (which translates to “we build together” in isiZulu) are leading the way in decolonial data practices (Orife et al. 2020). This is a grassroots NLP community creating machine translation models for African languages. The goal of this community is to ensure that Africans shape their own technological future where these technologies are developed and owned by Africans whilst focussing on human dignity, wellbeing, and equity through inclusive community building, open participatory research and multidisciplinarity. Rather than extracting data, Masakhane relies on local contributors, linguists, and speakers to build open, community-owned datasets.

Another example is the works of Te Mana Raraunga’s (Māori Data Sovereignty Network) and the International Indigenous Data Sovereignty Interest Group that led to the development of the CARE (Collective benefit, Authority to control, Responsibility, and Ethics) principles for Indigenous Data Governance (Carroll et al. 2020). These principles were aimed at ensuring that data practices reflect Indigenous worldviews. In terms of technology provision, there is also the Zenzeleni Networks (South Africa); a community-owned ISP providing affordable Internet in rural towns of South Africa, applying cooperative governance rather than corporate-driven models (Prieto-Egido et al. 2022).

These examples show that pluriversal approaches are not abstract ideals but active interventions that can reshape how technology is developed, governed, and deployed. Together, they signal a shift from communities being passive recipients of global AI coloniality to active producers of locally rooted, globally significant alternatives, positioning decoloniality as both a critique and a pathway to just, pluriversal technological futures.

## Methodology

This study employed a narrative literature review approach to explore and critically examine the scope, focus areas, and limitations of existing AI impact assessment tools and frameworks. The choice of narrative review here is shaped by the fact that it is preferable for a topic that is broad, complex, or emerging (Collins and Fauser 2005; Pae 2015). As Sukhera (2022) pointed out, narrative review is flexible, rigorous, and practical which are needed in this research. Coloniality is a complex social, historical, political, and epistemic issue, and impact assessments are diverse. The objective here was to identify how these tools conceptualise impact, which domains and ethical principles are prioritised, and to assess the extent to which they account for or neglect concerns related to coloniality and epistemic justice.

### Search strategy

The review was guided by the research question: *How do existing AI impact assessment tools address social, ethical, and political concerns, and to what extent do they engage with structural and decolonial critiques?* To address this question, a systematic search was conducted across a range of academic and grey literature sources. Academic databases, such as Scopus, Web of Science, and IEEE Xplore, were searched using combinations of keywords including: “AI impact assessment”, “algorithmic impact assessment”, and “AI, Impact Assessment, Risks”.

Additionally, grey literature, including policy reports, technical guidelines, non-governmental publications, and governmental and intergovernmental documents, was reviewed to capture non-academic contributions. Priority was given to documents from leading regulatory and advisory bodies, such as the European Commission, the UK’s ICO and the US National Institute of Standards and Technology (NIST), and relevant Chinese government guidelines.

### Inclusion and exclusion criteria

Documents were selected based on the following inclusion criteria: The primary focus of the document was the assessment of data and or AI systems (either through risk, impact, ethics, or accountability frameworks). The document contained explicit guidance, criteria, or methodologies for assessing the societal, ethical, legal, or technical impacts of AI. Documents published in English between 2015 and 2025 to capture the most relevant and recent developments in the field. Exclusion criteria included: articles or documents focussed solely on technical performance metrics without societal or ethical consideration, and duplicated or near-identical versions of documents published by the same institutions.

### Selection process

The initial search yielded approximately 122 documents. After reviewing titles and abstracts (or executive summaries, in the case of grey literature), 66 documents were shortlisted for full-text review. These were then assessed for relevance, originality, and conceptual contribution to the topic of AI impact assessment. Following the full-text review, 39 documents were selected as highly relevant to the research question. These final documents include a mix of academic publications, government guidelines, industry white papers, and NGO-produced toolkits. Each was analysed qualitatively for its stated objectives, scope, underlying ethical and legal principles, methodological approach, and evaluative criteria; see Table 1.

**Table 1:**
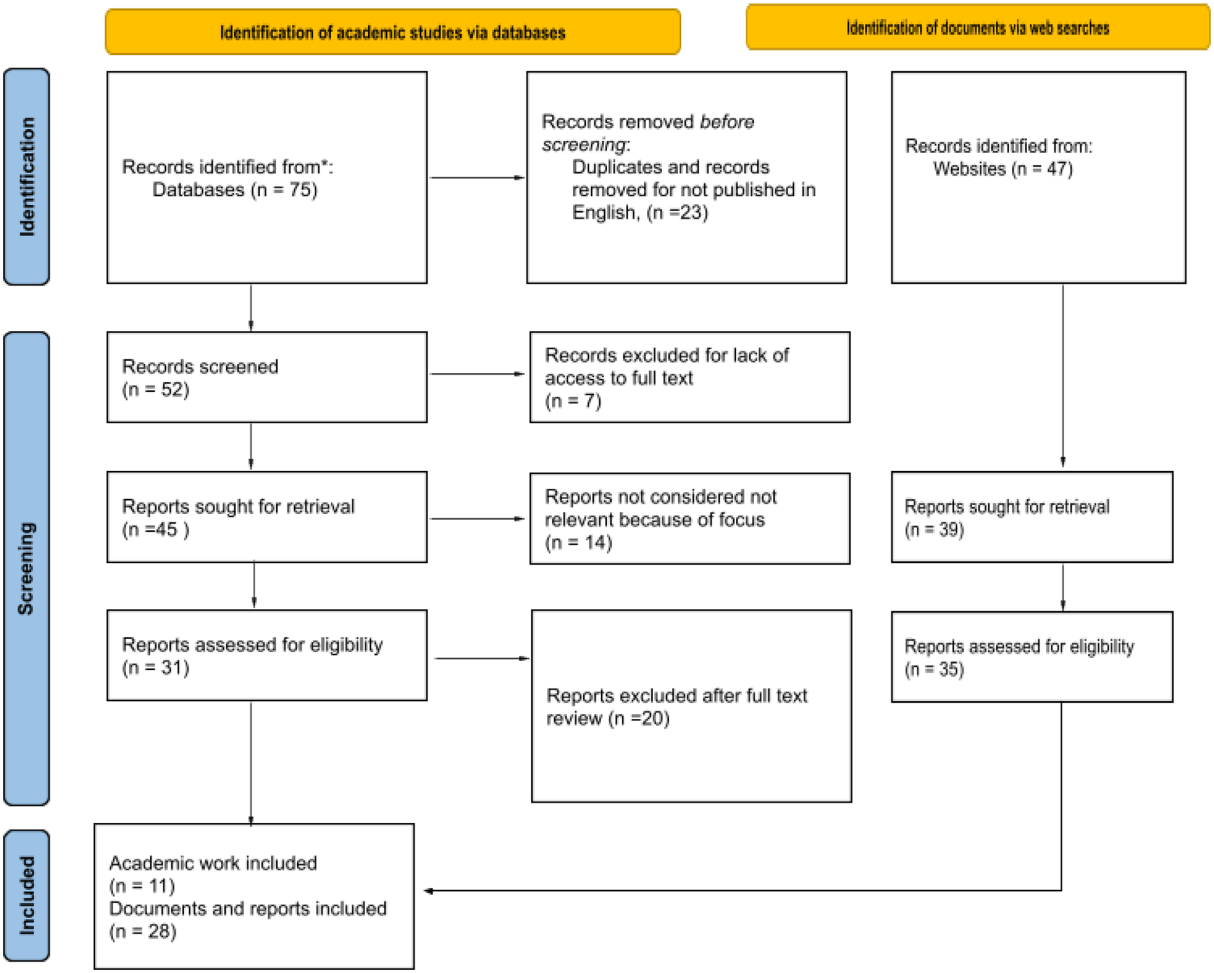
PRISMA flow diagram

### Data extraction and thematic synthesis

Key elements were extracted from each document, including: purpose of the tool or framework; ethical, legal, and policy domains addressed (e.g., privacy, fairness, human rights, transparency), geographic focus, presence or absence of references to structural, geo-political, or decolonial concerns. A thematic synthesis was then conducted to identify converging trends, dominant paradigms, and gaps across the selected documents. Particular attention was paid to patterns of omission, such as the lack of attention to power asymmetries, coloniality, or Global South epistemologies which informed the findings presented in the following sections of this paper.

## Key findings

Our review reveals a consistent emphasis on a set of normative and ethical concerns. Whilst these tools differ in structure, depth, and regulatory contexts, a number of recurring focus areas emerge, reflecting a shared global concern for aligning AI development with core social values. The most prominent amongst these is human rights, which forms a foundational pillar for many frameworks (Schmitt 2018; Mantelero 2018; HLEG 2019; Williams 2020; European Union Agency for Fundamental Rights 2020; Andrade and Kontschieder 2021; Council of Europe 2024; Ontario Human Rights Commission 2024). Whether through the lens of civil liberties, non-discrimination, or the prevention of harm, these assessment tools often seek to ensure that AI systems do not violate basic human dignity. Closely linked to this are data protection and privacy, reflecting both the risks of surveillance and the centrality of personal data in AI systems (Kaminski and Malgieri 2019; ICO 2020; Ivanova 2020; ICO 2025). Regulatory instruments like the EU’s GDPR have significantly shaped these emphases, especially within the European tools. This is regularly referred to as the Brussels effect (Bradford 2020).

Another dominant theme is ethics (ECP 2018; Devitt et al. 2020; Government Digital Service 2020; Brey et al. 2022; Deloitte 2024), sometimes explicitly articulated (e.g., “ethical and legal” evaluations) and at other times implied through principles such as responsibility (PWC 2019; PricewaterhouseCoopers 2019), accountability (AI Now Institute 2018; Reisman et al. 2018; DSIT, UK 2025; ISO 2025), safety (CAC 2024), and trust (NIST 2021; Winter et al. 2021; Zicari et al. 2021). These elements point to the concern around the moral obligations of AI developers, as well as the need for transparent and auditable systems that stakeholders can rely on (Gebru et al. 2020). On another note, trustworthiness, particularly, has become another way of capturing the concerns around transparency, reliability, explainability, and system robustness of AI systems.

Additionally, several frameworks address bias (UnBias 2018; Raji et al. 2020; ISO 2025; DSIT, UK 2025), fairness (UnBias 2018), and equality (EqualAI 2025), particularly in high-stakes domains, such as hiring (AI Now Institute 2018), policing, and recidivism prediction (Pennsylvania Board of Probation and Parole 2016; Oswald et al. 2018). These areas highlight the social risks of algorithmic discrimination and structural inequality being encoded and amplified through automated decision-making systems (Government of Canada 2024). Transparency and accountability are often positioned as ways of mitigating these risks, offering ways to scrutinise algorithmic processes and outcomes.

Furthermore, some tools focus on broader societal outcomes, such as human wellbeing (IEEE 2020), general social impacts (Ada Lovelace Institute 2020), and inclusion (EqualAI 2025), extending ethical evaluation beyond individual users to communities and social systems. Although these concerns are not always explicitly linked, their clustering across different tools suggests a normative convergence on certain high-level values meant to guide responsible innovation in AI.

Collectively, these focus areas point towards an emerging global consensus on what constitutes the responsible development and deployment of AI systems. They provide a valuable ethical and legal vocabulary for assessing risks and benefits, and they lay the groundwork for future normative elaborations, including those that might come from outside dominant geo-political spheres.

### The neglect of coloniality in existing impact assessment

One notable point from the above review is that all the existing AI IAs are silent on the deep-seated issue of coloniality and its enduring impact on contemporary technological systems. Whilst concerns, such as bias, fairness, transparency, accountability, and human rights, are increasingly mainstreamed in AI ethics and governance, a structured engagement with how historical and ongoing colonial power dynamics shape the development, deployment, and governance of artificial intelligence is missing. Existing assessments rarely question who defines risk, whose values are embedded in ethical frameworks, and which communities are consistently excluded from shaping AI futures. They assume governance and decision-making structures that align with western-like organisations but may not be suitable for some communities or regions. This results in impact assessments that may reinforce, rather than challenge, the very global inequities they are supposedly seeking to mitigate.

One clear example of this neglect is in the treatment of data governance. Whilst AI IAs emphasise privacy and data protection, they seldom address the extractive nature of data practices in the Global South, where communities often become passive data sources for models developed in the Global North. The asymmetrical flow of data, where African and other postcolonial contexts are mined for training data without meaningful benefit-sharing or representation in model development, is a form of what has been termed algorithmic colonialism (Birhane 2020). Yet, these dynamics are rarely made visible in the evaluation criteria of IAs.

Moreover, impact assessments often assume universal ethical standards without attending to local epistemologies, cultural values, or alternative ontologies. Concepts like fairness and autonomy are typically defined in Western liberal terms, with little or no room for relational ethics such as *ubuntu, Umunna* or indigenous notions of collective wellbeing and stewardship (Eke, Chintu and Wakunuma 2023a). This epistemic neglect sidelines valuable moral and philosophical traditions from Africa, Latin America, and other indigenous communities, further entrenching a near monolithic view of ethics and governance (Eke et al. 2025). For example, ubuntu’s perspective of autonomy is relational. The freedom to act is always embedded within social obligations and care for others. Risks to autonomy thus should include not only manipulative or coercive uses of AI (such as exploitative nudging and hidden surveillance) but also systems that weaken collective agency and community decision-making. Safeguarding autonomy under ubuntu is therefore about making sure that communities have the capacity to shape, question or reject technologies that do not align with their values. This is a narrative that is missing from western framing.

In essence, the neglect of coloniality in AI impact assessments reveals a fundamental gap in current global approaches to AI ethics and governance. For instance, this neglect also means the neglect of critical environmental footprints related to resource-intensive hardware production in the AI lifecycle. This omission entrenches unsustainable practices that undermine global efforts to address climate change and environmental degradation. Without confronting the colonial structures embedded in AI’s data, labour, infrastructure, and epistemology, existing impact assessments risk reproducing the very injustices they seek to prevent. Addressing this gap requires more than adding “diversity” as a checkbox; it calls for a paradigm shift that centres decolonial thinking, Global South perspectives, and epistemic justice in the very design and application of impact assessment tools. This informs the introduction of ‘decoloniality impact assessment’.

## Decoloniality impact assessment

Following the above examination of existing AI IA tools and frameworks, Decoloniality Impact Assessment (DIA) emerges as a crucial and necessary response to the identified gaps in terms of the coloniality problem.

DIA is a critical and context-sensitive evaluative approach that assesses the design, development, and deployment of technologies, particularly AI systems, in relation to their inherent colonial legacies, global power asymmetries, and epistemic injustices. Unlike traditional impact assessments that focus on ethics, legality, and technical risks, DIA goes further to interrogate how innovation practices may reinforce structural inequalities, marginalised vulnerable, indigenous and local people and knowledge systems, and perpetuate extractive and exploitative systems.

DIA is not only an extension of existing ethical frameworks; it is also a relational approach that aims to interrogate how innovation projects may be complicit in reproducing colonial power dynamics, both materially and epistemically. It calls attention to the fundamental values, principles, and beliefs that underpin much of contemporary technology development and deployment. This is particularly relevant when Global North actors engage with or operate in Global South contexts. In doing so, DIA invites a fundamental rethinking of whose knowledge and values matter, whose interests are served, and what it means to be “responsible” in innovation.

At its core, DIA reflects the intellectual and political traditions of decolonial thought. It highlights how innovation can operate as a continuation of colonial projects when it fails to recognise indigenous epistemologies, local cultural norms and principles, and alternative value systems. Unlike mainstream assessments that prioritise procedural ethics (e.g., fairness, transparency, and consent), DIA adds structural ethics, relational accountability, and historical justice. Furthermore, DIA addresses the geo-political power imbalances in AI development by highlighting the importance of contextual integrity, knowledge sovereignty, and fair benefit-sharing. It critiques the extractive tendencies of global AI infrastructure, from the mining of resources and labour exploitation to the harvesting of data from marginalised communities, whilst proposing a pluriversal, justice-oriented lens for evaluating innovation. By embedding concepts, such as relational ethics, ubuntu, and communal responsibility, DIA creates space for more meaningful participation, co-creation, and accountability in research and innovation.

DIA offers a way to assess not only the technical or ethical risks of innovation, but also its alignment with emancipatory values, social justice, and epistemic inclusion. It provides a tool for innovators, funders, policy-makers, and researchers to expand the boundaries of impact evaluation to account for the complex issues of history, power, and culture that contribute to shaping technological futures.

### AI innovation lifecycle approach to DIA

The nature of the coloniality problem requires DIA to be a granular and responsive model of responsible innovation; one that not only critiques colonial residues in AI, but also reimagines innovation in ways that are participatory, pluriversal, and deeply grounded in African and Indigenous values and principles. This is why, we suggest the AI innovation lifecycle approach as both a strategic and ethical imperative. The AI life cycle provides a structure to the DIA that reflects the understanding that coloniality and systemic imbalances are embedded at every stage of innovation; from ideation, design, development, use, to commercialisation. We also extend this to decommissioning and regulation of AI systems.

As highlighted in previous sections, coloniality manifests not only in the visible outcomes of AI (e.g., biased decisions, unfair deployments), but also in less visible processes, such as problem framing, design assumptions, development hierarchies, and regulatory capture. A lifecycle approach ensures that teams do not isolate ethical or decolonial concerns to the final stages (like deployment), but rather recognise that each phase may have different forms of power imbalance, marginalisation, or epistemic exclusion.

The influence of different actors in shaping the AI system varies throughout the life cycle: funders and policy-makers at ideation, engineers and designers at development, corporations at commercialisation, and regulators at the deployment stage through governance mechanisms. Each of these actors brings different worldviews, incentives, and responsibilities. A lifecycle-based structure allows DIA to hold each stakeholder group accountable according to their role, and ensures that decoloniality is integrated horizontally across actors and vertically across time.

Waiting until AI systems are deployed before asking decolonial questions is often too late. At that stage, much of the harm is already embedded, through biased data, colonial framings of problems, exploitative labour, or skewed power dynamics. A lifecycle assessment prompts early reflection and intervention, making it possible to reconfigure innovation pathways before harm is embedded.

AI systems and the context they are embedded in are never static. They evolve over time, are retrained with new data, and are reused in novel contexts. Structuring the DIA according to the lifecycle stages enables periodic reviews and iterative accountability, ensuring that developers revisit ethical and decolonial questions as systems grow. This is especially critical in contexts where sociopolitical realities change or where long-term impacts may only emerge over time. Post-deployment continuous risk monitoring is also critical, as a way to address the dynamic nature of socio-technical systems. Our proposed DIA steps, questions (in Box 1) for the different stages of the AI innovation cycle, suggested artefacts and indicators provide an idea of how DIA can be set up.

**Box 1:** Step-by-step Decoloniality Impact Assessment (DIA) questionnaire

***Step 1: Determine the need for DIA***	
● Is this AI system being developed, deployed, or tested in a context with historical or ongoing colonial, extractive, or power-imbalanced relations?	
● Will this system interact with or affect communities in the Global South or Indigenous/colonised populations?	
● Does the project involve cross-border data transfers, mineral resources, or international research partnerships?	
● Is there a risk of epistemic exclusion (i.e., privileging only Western/Global North knowledge systems or norms)?	
If yes to any, proceed with full DIA. However, if this is a smaller team, go through with step 2 and then organise at least one stakeholder consultation with affected community	
***Step 2: Description of the AI system***	
● What is the purpose and domain of the AI system (e.g. health, policing, and agriculture)?	
● Who are the main beneficiaries, funders, and developers?	
● Where will the system be developed, tested, and deployed?	
● What datasets, resources, infrastructures, or labour sources does it rely on?	
The steps below include key questions under the different stages of the AI innovation lifecycle as well as suggested practical tools, or records that can be during the AI lifecycle to capture decisions, power relations, and community engagement. They make decoloniality visible and documentable. These are what we call artefacts. They also include indicators which are measurable dimensions (quantitative or qualitative) that help assess the presence, degree, or risk of coloniality in AI systems	
***Step 3: Ideation stage***	
Key questions:	
● Whose problem is being solved? Who defined the problem?	
● Were local communities, Indigenous knowledge holders, or Global South actors involved in framing the need?	
● Are alternative problem framings or solutions being considered?	
● Are there power imbalances in the agenda-setting process (e.g., foreign donors defining priorities)?	
Suggested artefacts:	
● Positionality & power mapping card: a structured worksheet for identifying which actors hold power and whose perspectives are marginalised	
Suggested indicator:	
● Data Sovereignty Index: measures the proportion of local institutions involved in the data value chain	
***Step 4: Design stage***	
● Whose values, ethics, and assumptions are embedded in the design (e.g., privacy—individual or collective)?	
● Were local or indigenous epistemologies and cultural norms consulted or considered in design decisions?	
● Were gender, class, race, and disability lenses applied to ensure inclusive and non-extractive design?	
● Are data practices (collection, annotation, and storage) aligned with data justice and digital sovereignty principles?	
● Are resource implications (e.g., use of rare-earth minerals or energy) assessed through a decolonial lens?	
Suggested Artefacts:	
● Design reflexivity log: A log where design teams document their decisions, assumptions, and trade-offs during the AI system’s design. This will capture the process of making design choices and trade-offs	
Suggested indicator:	
● Inclusivity Index: a metric that measures the diversity and representativeness of both datasets and design teams	
***Step 5: Development stage***	
● Who is developing the AI system? Are Global South experts and communities represented in the core team?	
● Are data labelling and annotation outsourced to low-wage workers in exploitative conditions?	
● Are there mechanisms for fair compensation, safe working conditions, and voice for annotators and low-skilled contributors?	
● Are datasets sourced with full consent, adequate context, and recognition of data subjects’ rights?	
● Are African or Indigenous languages and contexts represented in the data and models?	
Suggested Artefact:	
● Testing & Validation Log: record of how the system was tested across diverse sub-groups (including minority or marginalised groups)	
Suggested indicator:	
● Performance Equity Index: ratio of model accuracy/performance across majority vs. minority groups (measures equity in outcomes)	
***Step 6: Use and deployment stage***	
● Will this AI system reinforce existing inequalities (e.g., through biased predictions or exclusions)?	
● Are deployment contexts (e.g., lack of legal safeguards, low data literacy) properly understood and addressed?	
● Are communities informed and meaningfully involved in decisions about the system’s use?	
● Are there accessible feedback mechanisms for affected users or groups?	
● Does the system enable local capacity-building or just perpetuate dependency?	
Suggested artefact:	
● Register of relational community consent (documenting ongoing, collective, and revocable consent): a document that records collective, ongoing, and revocable consent from communities whose data, knowledge, or labour is used in an AI system	
Suggested indicator:	
● Benefit-sharing index: metric assessing the degree to which local communities contributing data, labour, or knowledge receive tangible benefits	
***Step 7: Commercialisation stage***	
● Who owns the AI system, and who profits from it?	
● Are benefit-sharing mechanisms in place for local communities or data-contributing populations?	
● Is intellectual property governed in a way that supports knowledge sovereignty?	
● Are local economic systems and informal economies respected and included?	
● Does commercial strategy encourage monopolisation or platform extractivism?	
Suggested artefact:	
● Market impact assessment log: record of anticipated effects on local economies, including risks of displacing local industries or traditional practices	
Suggested indicator:	
● Market Equity Risk score: likelihood and scale of harm to local markets or livelihoods due to the introduction of commercialised AI products (e.g., job displacement, dependency on foreign vendors)	
***Step 8: Governance and regulation stage***	
● Are regulatory frameworks aligned with the political, legal, and cultural contexts of the regions where the AI is deployed?	
● Are unique African/Global South community-based governance models involved?	
● Does governance address structural inequalities or simply enforce procedural compliance?	
● Are Indigenous principles (e.g. ubuntu, intergenerational justice) integrated into governance?	
● Does the regulatory approach challenge or reproduce technocratic dominance and Western epistemic authority?	
Suggested artefact:	
● Community Oversight Charter: Governance framework that defines how communities participate in oversight, monitoring, and decision-making processes	
Suggested indicator:	
● Regulatory Alignment Index: the extent to which governance frameworks respect local sovereignty (local laws, cultural norms) whilst aligning with international human rights and AI norms	
***Step 9: Assessment results (traffic light system)***	
For each stage, assign a status based on evidence and responses:	
Green: if positive on all questions; aligned with decolonial principles	
Amber: If negative on at least one question; partial compliance and requires mitigation or improvement	
Red: If negative on more than half of the questions; misaligned and presents significant coloniality risks	
***Step 10: Periodic review and iteration***	
● Has a timeline for reviewing DIA outcomes been established?	
● Are there mechanisms to update and re-evaluate impact as the AI system evolves?	
● Are community stakeholders involved in the review process?	
● Has the system adapted in response to feedback from users and impacted groups?	

### Key stakeholders and conditions for successful implementation

A central aspect of the proposed DIA is multi-stakeholder inclusion which is not particularly different from other IAs. However the emphasis here are the voices of historically marginalised groups. The implementation of DIA requires participation from different stakeholder categories; developers and designers to provide technical details and engage in reflexive design, project managers and funders to embed DIA in project workflows, budgets and timelines, social scientists to guide normative and power-sensitive analysis, local communities and users to share lived experiences, cultural insights, and contextual feedback, policy-makers and regulators to align DIA with compliance frameworks and ensure accountability, civil society and advocacy groups to monitor fairness, transparency, and the integrity of the DIA process, historians and decolonial scholars to contextualise historical power dynamics and offer counter-narratives and labour representatives to represent the workers like data labellers whose conditions are often excluded from ethical oversight. It is also important to mention that ensuring the above tasks may be unfeasible for some organisations (e.g., a startup will likely lack resources to bring in policy-makers, regulators, and social scientists to perform the assessments). These organisations can go through the first two steps of our proposed approach in Box 1 and then focus on running low-cost participatory consultations with affected communities and embedding simple but impactful safeguards, such as contractual clauses on benefit-sharing and community consent. They can document decisions using basic reporting logs and set clear thresholds for when a more comprehensive DIA is needed (e.g., sensitive health data or when vulnerable groups are involved). This lightweight pathway ensures accountability and inclusivity without overwhelming smaller teams, whilst still aligning them with decolonial and responsible AI governance principles.

Additionally, to ensure the meaningful implementation of DIA, some enabling conditions are necessary. The first is the integration of decolonial ethics as an integral part of capacity-building and training on AI literacy, since this is already a regulatory requirement in most AI governance frameworks (Cabral 2025). As an emerging concept in AI, many people are yet to be acquainted with the meaning and implications of decoloniality. It is also important to institutionalise the embedding of DIA into research and development pipelines, procurement, and evaluation mechanisms. Another necessary condition is the provision of resources and funding to support inclusive participation. This should also be supported by clear policy integration through national or regional digital governance frameworks that can ensure implementation.

The implementation of DIA is not simply a new form of impact management. It is a call to reframe how AI innovation is done. By embedding a structured, participatory, and lifecycle-sensitive process that addresses coloniality, we can begin to reimagine technological futures rooted in justice, pluriversality, and sovereignty. The benefits of doing DIA are demonstrated with hypothetical use cases in Box 2.

**Box 2:** Two hypothetical cases to illustrate the potential benefits of doing AI impact assessments with and without DIA

***Case 1 public sector: health AI in a low-income country***	
A national health ministry partners with an international tech company to design, develop and deploy an AI tool for predicting epilepsy risk using neuroimaging and neurochemical biomarkers. Doing an impact assessment on this system…	
Without a DIA:	
• Data can still be primarily sourced from hospitals and clinics in urban areas, with minimal representation of rural patients	
• Datasets can be transferred to the international tech company cloud system affecting the control and ownership of the data	
• No consultation may be held with communities contributing neurochemical data	
• Errors can disproportionately affect rural populations whose dialects and clinical profiles were excluded	
• Commercial rights and IP may be retained entirely by the foreign partner and the system can become too expensive for widespread local adoption	
• And finally communities may lose trust in the tool, with some refusing to share data further	
With a DIA:	
• Co-creation workshops can identify local priorities	
• A register of relational community consent can record community approval, with the option to revoke if harms arise	
• Benefit-sharing clause in the contract can ensure 15% of licencing revenue is reinvested in local epilepsy care and training of local professionals	
• Data Sovereignty Index can help to ensure the retention of ownership and control of the data	
• Performance Equity Index is tracked to ensure diagnostic accuracy across urban and rural sub-groups	
Result: Greater trust, broader adoption, improved patient outcomes, and local research capacity-building	
***Case 2 private sector: startup developing agricultural AI***	
A small-to-medium African agritech startup builds an AI system to predict soil fertility and optimise crop yields	
Without a DIA:	
• The model may be trained largely on open-access dataset (of satellite imagery from many parts of the world)	
• African farmers’ local knowledge about soil practices may not be integrated	
• IP may be sold to a multinational agribusiness, which commercialises the product at a cost inaccessible to small scale farmers	
• Local farmers become dependent on foreign-controlled platforms, displacing indigenous practices	
• With a DIA:	
• A Design Reflexivity Log can document trade-offs, including choices to prioritise integrating local soil samples despite resource constraints	
• A Community Oversight Charter can ensure farmers’ unions are consulted at key milestones	
Result: The startup retains legitimacy, secures regional adoption, and strengthens local ownership of agricultural innovation	

### Embedding DIA into existing IAs

Having demonstrated that coloniality is a key issue that current approaches to ethical and related issues of AI currently neglect, the question is how best to overcome this limitation of extant AI impact assessments. There is no simple and unambiguous answer to this question. However, one can distinguish between different strategies of approaching it. On the one hand, it is conceivable to position DIA as another type of impact assessment that complements existing impact assessment, such as environmental impact assessment, human rights impact assessment, and many others. In the context of AI, this approach would suggest that an individual or organisation concerned about AI impacts could add such a stand-alone DIA to the array of assessments they can apply to a system or application at hand. This would have the advantage of methodological purity, as it would allow focussing exclusively on questions of coloniality as described above. However, that is not how we are positioning DIA.

An alternative approach would be to integrate and embed DIA into existing AI impact assessments. The advantage would be that a potential user of the impact assessment would not need to do additional research to find out about the relevance of coloniality and relevant aspects would be integrated into the findings and results of the AI impact assessment. In light of the rapidly proliferating number of AI impact assessments and their increasing importance with regards to demonstrating legal compliance, we suggest that the latter option of embedding DIAs into other impact assessments is more likely to raise the profile of questions of coloniality and thus to have the decolonial consequences that the discussion of decoloniality aims for. In addition to avoiding duplication, integration of decoloniality into proven governance processes (e.g., risk management according to international standards) will help to improve efficiency. We therefore outline how such embedding might be put in practice.

In this paper, therefore, we are proposing the integration of DIA into existing IAs. However, it is important to note distinct features that separate current HRIA/FRIA practices and emerging ISO/IEC standards from DIA. ISO/IEC 42001 (AI management systems) and ISO/IEC 23894 (AI risk management) have structured governance and generic risk processes; however, they do not explicitly address colonial legacies, power asymmetries, or epistemic justice. The same with FRIA under the EU AI Act that focuses on *fundamental rights* risks for high-risk uses; it does not require decolonial analysis, community self-determination, or benefit-sharing. As regards HUDERIA (Council of Europe), it targets human rights, democracy and rule of law and it is strong on rights/risk structure but not designed to address coloniality of data nor does it mandate reparative design. DIA, on the other hand, focuses on decolonial theory and practice (e.g., power/knowledge, data sovereignty, and reparative governance) to complement and not replace these instruments. See more details of these distinct features in Table 2.

**Table 2:** Examples of what DIA adds to existing IAs

Requirement	What ISO/FRIA/HUDERIA already has	Complementarity of DIA
Scope	ISO sets organisation-wide AI governance/risk processes; FRIA triggers for high-risk and public uses; HUDERIA guidance for rights, democracy and rule-of-law risks	Applies to all stages of AI systems lifecycle irrespective of risk level especially when projects touch historically marginalised communities
Problem framing	Generic impact and risks; rights	Analysis of colonial legacies with explicit mapping of power imbalances and control
Epistemic justice	Not explicit	Requires pluriversal perspectives; knowledge sources and data labels
Consent/autonomy	FRIA/HUDERIA expect stakeholder engagement; consent often framed at data subject level	Adds collective/community consent
Data provenance & sovereignty (data and infrastructure)	ISO/FRIA check lawfulness, quality, bias; data sovereignty not required	Requires audits for coloniality of data (extractive practices, context loss), enforces data and infrastructure sovereignty (local storage, access terms) and culturally bounded data categories
Language and cultural rights	All have bias/fairness checks; no duty to support minority languages beyond performance metrics	Requires plans for linguistic inclusion, protection of cultural IP, and community control over cultural models
benefit-sharing	Not explicitly required	Requires equitable value-sharing (including local hiring/skills transfer) tied to design and deployment approvals
Procurement & local capacity	Supplier oversight and process controls (ISO 42001)	Encourages local suppliers or local partnerships; mandating training and institutional capacity-building as part of the innovation lifecycle

However, one challenge of the approach to integrating DIA into other types of impact assessments is that there is a multitude of such assessments, including specialised impact assessments for AI as our review shows. Furthermore, these impact assessments, once published and used, are not always easy to modify. However, the field of AI impact assessments is still relatively novel and in flux and there is no fully established best practice standard. We therefore believe that our proposal to integrate DIA into other impact assessments is feasible and we hope that this article can support such efforts. Many of existing IAs are non-exhaustive and, in several cases, acknowledge the need to align to specific case needs. This adaptability and agility in IAs offer the opportunity for the intended integration of DIA.

Drawing on a systematic review of AI impact assessments (Stahl et al. 2023), it is easy to see in which way and at which stages issues and questions related to coloniality can be integrated into the generic impact assessment structure developed by the paper. Stahl et al. (2023) propose that the owner of the AI impact assessment starts by defining the AI system, including its purpose, technical specifications, and benefits. On this basis the owner then decides whether the system is expected to have social or ethical impacts. If such impacts are expected, then the next step is the determination of stakeholder categories. This is the first step where DIA can play a role. The inclusion of victims of coloniality and otherwise exploited indigenous communities can change the makeup of the stakeholder representatives who are selected on the basis of stakeholder categories. The next place where DIA is of relevance is in the identification of possible issues and metrics used to assess them. The issues of coloniality as described in Sect. 2 above can complement the existing issues, such as human rights, safety, or environmental impacts. According to Stahl et al. (2023), the AI impact assessment then needs to be integrated into the broader impact assessment regime of the organisation which may include existing impact assessments, such as data protection impact assessments or other risk management tasks. Finally, the impact assessment needs to be used to develop mitigation strategies and an action plan which, where appropriate, will be published and laid open to scrutiny. This structure is iterative and needs to be repeated at regular intervals or where new technical developments or unexpected consequences arise. In this respect, O’Neale et al. (2025) provides ten guidelines on how to decolonise algorithm systems, such as articulating a set of foundational principles, identifying and engaging meaningfully with key partners, considering different scopes of the algorithm, ensuring that algorithmic transparency is often compatible with and facilitated by open-source software, including local developers or practitioners, embedding identifying, reporting and tracking issues, considering the implications of the algorithm for both individual and collective privacy, determining baselines, documenting codes, and building capacities for individuals and collectives.

### DIA and HUDERIA

The above overview indicates that the integration of a DIA into the overall logic of AI impact assessments is possible and can be seen as supplementing existing logics and structures of impact assessments. It is, however, also quite abstract. For this reason, we now look in some more detail at a particular impact assessment to explore where and how DIA could be integrated. We chose the “Methodology for the Risk and Impact Assessment of Artificial Intelligence Systems from the Point of View of Human Rights, Democracy and the Rule of Law (HUDERIA Methodology)” proposed by the Committee on Artificial Intelligence of the Council of Europe (Council of Europe 2024). The HUDERIA methodology is a suitable example for our purposes, because it is not confined to a particular technology, application, or organisation.

The Council of Europe (CoE) describes its HUDERIA framework as a methodology for risk and impact identification, assessment, prevention, and mitigation applicable to a variety of AI technologies and applications (Council of Europe 2024). It furthermore aims to promote compatibility and interoperability between existing and future standards and frameworks from organisations, such as ISO, IEEE, ITU as well as NIST and EU AI Act requirements. It is therefore uniquely suited for our purpose of demonstrating the relevance of DIA, as it does not represent a specific impact assessment, but aims to offer a broader framework that is applicable across many impact assessments.

HUDERIA consists of four elements: a Context-Based Risk Analysis (COBRA), the Stakeholder Engagement Process (SEP), a Risk and Impact Assessment (RIA), and the Mitigation Plan (MP). Whilst the DIA seems most closely aligned to the third element, the RIA, it is in fact relevant to all elements. The COBRA stage of HUDERIA aims to carry out the preliminary background research needed to inform subsequent risk factor identification and impact mapping activities. This is done by mapping risk factors in relation to three aspects of the AI system: the application context, the development context, and the deployment context. Questions of coloniality may arise in all three of these contexts, but they may look different and have different implications. The COBRA analysis of risk factors matches to some extent the lifecycle approach for DIA that we introduced earlier. The main point here is that DIA can broaden the understanding of risks of AI arising due to its focus on questions of coloniality. COBRA determines the risk levels in the step following the risk identification. This is done by determining the scale, scope, reversibility, and probability of expected risks. Whilst risks related to coloniality may not be qualitatively different from other risks, such as those related to human rights, this is a step that requires further research on appropriate ways of determining, measuring, and possibly quantifying such risks. One issue at stake here is that classical quantification of risks often makes an assessment based on a population. This often leads to quantification based on the average distribution, which may penalise minorities. Hence, quantification of these risks may require means to assess impact not only at the level of the whole population, but also differential impact in sub-groups.

The second step of HUDERIA is that of stakeholder engagement. This is one point where DIA is likely to significantly strengthen HUDERIA. As part of the stakeholder analysis, HUDERIA posits the need for “positionality reflection” which calls for the identification of the limitations of HUDERIA users’ perspectives and identifying missing viewpoints (Council of Europe 2024). To some degree, this inclusion of missing viewpoints is at the heart of DIA. Questions of coloniality are typically not at the top of the minds of individuals who have no history of being colonised which can lead to the exclusion of the viewpoints of those with a colonial history. The SEM step of HUDERIA requires not just the identification of missing stakeholders and perspectives but also the choice of an appropriate method of engagement. Again, experience of engagement with stakeholders related to coloniality can inform the implementation of HUDERIA.

The third step, RIA, calls for the detailed evaluation and impacts of AI within the lifecycle of the system. This is based on the earlier COBRA analysis and involves the stakeholder identified in the SEM stage. At this point, the earlier COBRA descriptions of possible risks are empirically investigated in more detail with the aim to collect reliable information on their scale, scope, reversibility, and probability. Whilst we cannot provide detailed guidance on how to do this with coloniality-related risks, we reiterate that the incorporation of DIA into this step will lead to a more in-depth understanding of AI risks. Specific pathways of integration will now be the necessary next step for DIA. This will position DIA as a viable norm that can assist institutions in circumventing "decolonial-washing" and authentically redirecting innovation cycles towards justice, pluriversality, and the sovereignty of knowledge.

The final step of HUDERIA is the mitigation of the risks. This includes the formulation of mitigation measures, creation of a mitigation plan, and potentially setting up access to remedy. HUDERIA emphasises the importance of legal obligation in the mitigation stage, but is by no means limited to them. The methodology includes a suggestion on how to prioritise mitigation options that includes four levels: avoid, mitigate, restore, and compensate. Questions of coloniality are broad and based on centuries of history. Mitigating coloniality-based risks cannot be the sole responsibility of an individual organisation that develops AI systems. Rather, ongoing political discussions about the consequences of colonialism and possible reparations for slavery and other crimes of colonialism will form part of this discussion. This implies that the mitigation will need to consider the broader socio-economic and political ecosystem within which an AI system is embedded to find suitable mechanisms for mitigation. This, in turn, implies that HUDERIA mitigation is not exclusively a technical exercise. The inclusion of DIA highlights that HUDERIA needs to be understood at least partly, as a contribution to the broader political discourse around AI, how new technologies are created and diffused, and which responsibility structures exist or need to be developed to provide a governance structure that allows a beneficial use of these technologies.

## Conclusion

In this article, we have argued that questions of coloniality and decoloniality are of potentially high importance for AI development and deployment. Despite this importance, there is little evidence to suggest that decoloniality has found its way into existing AI impact assessments. We therefore propose the idea of a decoloniality impact assessment to overcome this current blind spot. We then go on to outline the principles and content of DIA and discuss how the ideas can be integrated into existing impact assessments, using the example of HUDERIA.

The answer to our research question of how existing AI impact assessment tools address social, ethical, and political concerns, and to what extent do they engage with structural and decolonial critiques is relatively straightforward: they address a broad range of concerns, but not those specifically related to decoloniality. We believe that this insight is of importance to the broader AI and AI governance discourse. Given that AI technologies, tools, and techniques are of a potentially global nature, this state of affairs is not acceptable. Moreover, the current distribution of power and resources in AI with a strong concentration in very few countries, and organisations raises the spectre of a new round of colonialism, this time in the form of data, information, or AI colonialism. It risks the further exploitation of communities who often still suffer from past experience of colonialism.

By proposing DIA and its possible integration into other assessment methods, we suggest a practical and implementable way of addressing this issue. This is not a simple, straightforward and linear solution. Problems of coloniality pervade societies and the global political discourse. Coloniality in AI is only one aspect of this. However, in light of the continuously growing importance of AI and its reach across all aspects of the economy and society, we believe that an explicit emphasis on decoloniality is called for and should be an integral part of AI design, development, deployment, application, and governance.

## Data Availability

The final list of the documents reviewed and analysed can be accessed openly through the University of Nottingham data repository with this DOI (10.17639/nott.7591).
